# (*E*)-*N*-(2,3,4-Trimeth­oxy-6-methyl­benzyl­idene)naphthalen-1-amine

**DOI:** 10.1107/S160053680803910X

**Published:** 2008-12-10

**Authors:** Cheng-Yun Wang

**Affiliations:** a Department of Chemistry and Chemical Engineering, Weifang University, Weifang 261061, People’s Republic of China

## Abstract

In the title compound, C_21_H_21_NO_3_, the dihedral angle between the naphthalene ring system and the substituted benzene ring is 55.7 (2)°. The mol­ecules are linked into a zigzag chain running along the *b* axis by C—H⋯O hydrogen bonds.

## Related literature

For a related structure, see: Zhang (2008 ▶).
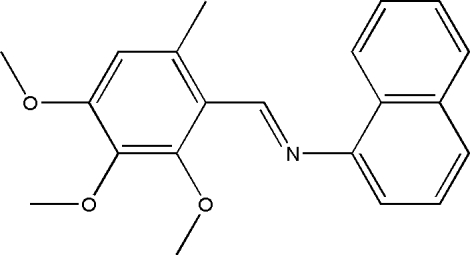


## Experimental

### 

#### Crystal data

C_21_H_21_NO_3_
*M* *_r_* = 335.39
Orthorhombic, 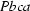

*a* = 10.9225 (14) Å
*b* = 14.7630 (16) Å
*c* = 22.514 (2) Å
*V* = 3630.3 (7) Å^3^
*Z* = 8
Mo *K*α radiation
μ = 0.08 mm^−1^
*T* = 298 (2) K
0.23 × 0.19 × 0.08 mm

#### Data collection

Bruker SMART CCD area-detector diffractometer
Absorption correction: multi-scan (*SADABS*; Bruker, 1997 ▶) *T*_min_ = 0.981, *T*_max_ = 0.994
17242 measured reflections
3195 independent reflections
1918 reflections with *I* > 2σ(*I*)
*R*_int_ = 0.071

#### Refinement

*R*[*F* ^2^ > 2σ(*F* ^2^)] = 0.059
*wR*(*F* ^2^) = 0.166
*S* = 1.07
3195 reflections
226 parameters
H-atom parameters constrained
Δρ_max_ = 0.18 e Å^−3^
Δρ_min_ = −0.25 e Å^−3^

### 

Data collection: *SMART* (Bruker, 1997 ▶); cell refinement: *SAINT* (Bruker, 1997 ▶); data reduction: *SAINT*; program(s) used to solve structure: *SHELXS97* (Sheldrick, 2008 ▶); program(s) used to refine structure: *SHELXL97* (Sheldrick, 2008 ▶); molecular graphics: *SHELXTL* (Sheldrick, 2008 ▶); software used to prepare material for publication: *SHELXTL*.

## Supplementary Material

Crystal structure: contains datablocks global, I. DOI: 10.1107/S160053680803910X/ci2730sup1.cif

Structure factors: contains datablocks I. DOI: 10.1107/S160053680803910X/ci2730Isup2.hkl

Additional supplementary materials:  crystallographic information; 3D view; checkCIF report

## Figures and Tables

**Table 1:** Hydrogen-bond geometry (Å, °)

*D*—H⋯*A*	*D*—H	H⋯*A*	*D*⋯*A*	*D*—H⋯*A*
C13—H13⋯O3^i^	0.93	2.56	3.489 (4)	178

Symmetry code: (i) 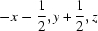.

**Fig. 1.**
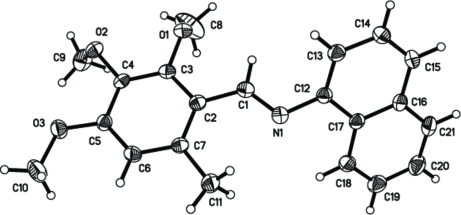
The molecular structure of the title compound, showing 30% probability displacement ellipsoids.

## supplementary crystallographic information

### Comment

The preparation, properties and applications of Schiff bases are important in the development of coordination chemistry. In this paper, the crystal structure of the title compound is reported.

Bond lengths and angles of the title molecule (Fig.1) agree with those observed in a related compound, (*E*)-*N*-(2,3,4-trimethoxy-6-methylbenzylidene)aniline (Zhang, 2008). The dihedral angle between the naphthalene ring system and the substituted benzene ring is 55.7 (2)°. One of the methoxy groups is coplanar (C10—O3—C5—C6 = 2.4 (4)°) with the attached ring whereas the other two methoxy groups are twisted (C8—O1—C3—C4 = -78.3 (4)° and C9—O2—C4—C3 = 109.1 (3)°).

The molecules are linked into a zigzag chain running along the *b* axis by C—H···O hydrogen bonds (Table 1).

### Experimental

A mixture of 1-naphthylamine (0.715 g, 5 mmol) and 2,3,4-trimethoxy-6-methylbenzaldehyde (1.04 g, 5 mmol) in ethanol (30 ml) was refluxed for 2 h. After cooling, the precipitate obtained was filtered and dried. The crude product was (20 mg) was dissolved in ethanol (20 ml) and the solution was filtered to remove impurities, and then left for crystallization at room temperature. Single crystals suitable for X-ray crystal structure determination were obtained after a week.

### Refinement

H atoms were positioned geometrically (C-H = 0.93–0.96 Å) and refined as riding with *U*_iso_(H) = 1.2*U*_eq_(C) or 1.5 *U*_eq_(methyl C).

### Crystal data

**Table:**

C_21_H_21_NO_3_	*F*(000) = 1424
*M**_r_* = 335.39	*D*_x_ = 1.227 Mg m^−^^3^
Orthorhombic, *P**b**c**a*	Mo *K*α radiation, λ = 0.71073 Å
Hall symbol: -P 2ac 2ab	Cell parameters from 3424 reflections
*a* = 10.9225 (14) Å	θ = 2.5–25.3°
*b* = 14.7630 (16) Å	µ = 0.08 mm^−^^1^
*c* = 22.514 (2) Å	*T* = 298 K
*V* = 3630.3 (7) Å^3^	Plate, light yellow
*Z* = 8	0.23 × 0.19 × 0.08 mm

### Data collection

**Table:**

Bruker SMART CCD area-detector diffractometer	3195 independent reflections
Radiation source: fine-focus sealed tube	1918 reflections with *I* > 2σ(*I*)
graphite	*R*_int_ = 0.071
φ and ω scans	θ_max_ = 25.0°, θ_min_ = 1.8°
Absorption correction: multi-scan (*SADABS*; Bruker, 1997)	*h* = −12→8
*T*_min_ = 0.981, *T*_max_ = 0.994	*k* = −17→15
17242 measured reflections	*l* = −26→25

### Refinement

**Table:**

Refinement on *F*^2^	Primary atom site location: structure-invariant direct methods
Least-squares matrix: full	Secondary atom site location: difference Fourier map
*R*[*F*^2^ > 2σ(*F*^2^)] = 0.059	Hydrogen site location: inferred from neighbouring sites
*wR*(*F*^2^) = 0.166	H-atom parameters constrained
*S* = 1.07	*w* = 1/[σ^2^(*F*_o_^2^) + (0.0824*P*)^2^] where *P* = (*F*_o_^2^ + 2*F*_c_^2^)/3
3195 reflections	(Δ/σ)_max_ = 0.001
226 parameters	Δρ_max_ = 0.19 e Å^−^^3^
0 restraints	Δρ_min_ = −0.25 e Å^−^^3^

### Special details

**Table:**

Geometry. All e.s.d.'s (except the e.s.d. in the dihedral angle between two l.s. planes) are estimated using the full covariance matrix. The cell e.s.d.'s are taken into account individually in the estimation of e.s.d.'s in distances, angles and torsion angles; correlations between e.s.d.'s in cell parameters are only used when they are defined by crystal symmetry. An approximate (isotropic) treatment of cell e.s.d.'s is used for estimating e.s.d.'s involving l.s. planes.
Refinement. Refinement of *F*^2^ against ALL reflections. The weighted *R*-factor *wR* and goodness of fit *S* are based on *F*^2^, conventional *R*-factors *R* are based on *F*, with *F* set to zero for negative *F*^2^. The threshold expression of *F*^2^ > σ(*F*^2^) is used only for calculating *R*-factors(gt) *etc*. and is not relevant to the choice of reflections for refinement. *R*-factors based on *F*^2^ are statistically about twice as large as those based on *F*, and *R*- factors based on ALL data will be even larger.

### Fractional atomic coordinates and isotropic or equivalent isotropic displacement parameters (Å^2^)

**Table:**

	*x*	*y*	*z*	*U*_iso_*/*U*_eq_	
N1	0.1079 (2)	0.32182 (15)	0.65758 (10)	0.0437 (6)	
O1	−0.20286 (18)	0.26652 (12)	0.57093 (9)	0.0494 (6)	
O2	−0.34730 (17)	0.11513 (12)	0.58640 (8)	0.0463 (5)	
O3	−0.29401 (19)	−0.00497 (13)	0.67264 (8)	0.0521 (6)	
C1	0.0044 (3)	0.30050 (17)	0.63498 (12)	0.0404 (7)	
H1	−0.0271	0.3393	0.6063	0.048*	
C2	−0.0689 (2)	0.22122 (17)	0.64990 (12)	0.0374 (7)	
C3	−0.1715 (3)	0.20404 (17)	0.61366 (11)	0.0360 (7)	
C4	−0.2445 (2)	0.12749 (19)	0.62117 (11)	0.0354 (7)	
C5	−0.2172 (3)	0.06808 (17)	0.66764 (12)	0.0382 (7)	
C6	−0.1196 (3)	0.08606 (19)	0.70516 (12)	0.0429 (8)	
H6	−0.1040	0.0467	0.7365	0.051*	
C7	−0.0440 (3)	0.16116 (18)	0.69743 (12)	0.0397 (7)	
C8	−0.1737 (5)	0.2453 (3)	0.51222 (15)	0.1067 (17)	
H8A	−0.1021	0.2076	0.5114	0.160*	
H8B	−0.1579	0.3001	0.4905	0.160*	
H8C	−0.2409	0.2136	0.4943	0.160*	
C9	−0.3371 (3)	0.0441 (2)	0.54325 (14)	0.0654 (10)	
H9A	−0.2605	0.0495	0.5227	0.098*	
H9B	−0.4032	0.0487	0.5153	0.098*	
H9C	−0.3408	−0.0136	0.5629	0.098*	
C10	−0.2751 (4)	−0.0653 (2)	0.72126 (16)	0.0843 (13)	
H10A	−0.1990	−0.0969	0.7160	0.126*	
H10B	−0.3410	−0.1083	0.7229	0.126*	
H10C	−0.2725	−0.0315	0.7576	0.126*	
C11	0.0612 (3)	0.1741 (2)	0.73974 (14)	0.0616 (10)	
H11A	0.1370	0.1649	0.7190	0.092*	
H11B	0.0548	0.1313	0.7717	0.092*	
H11C	0.0590	0.2345	0.7555	0.092*	
C12	0.1640 (3)	0.40259 (17)	0.63702 (12)	0.0367 (7)	
C13	0.1017 (3)	0.48289 (18)	0.63178 (13)	0.0450 (8)	
H13	0.0191	0.4855	0.6415	0.054*	
C14	0.1619 (3)	0.56103 (19)	0.61192 (15)	0.0539 (9)	
H14	0.1181	0.6148	0.6086	0.065*	
C15	0.2820 (3)	0.56022 (19)	0.59751 (14)	0.0530 (8)	
H15	0.3198	0.6130	0.5843	0.064*	
C16	0.3507 (3)	0.47867 (18)	0.60249 (13)	0.0423 (7)	
C17	0.2925 (3)	0.39934 (17)	0.62421 (11)	0.0375 (7)	
C18	0.3634 (3)	0.32002 (19)	0.63141 (13)	0.0463 (8)	
H18	0.3272	0.2681	0.6469	0.056*	
C19	0.4841 (3)	0.3187 (2)	0.61596 (15)	0.0582 (9)	
H19	0.5294	0.2659	0.6209	0.070*	
C20	0.5401 (3)	0.3956 (2)	0.59289 (15)	0.0613 (9)	
H20	0.6220	0.3934	0.5818	0.074*	
C21	0.4760 (3)	0.4741 (2)	0.58642 (14)	0.0562 (9)	
H21	0.5148	0.5252	0.5713	0.067*	

### Atomic displacement parameters (Å^2^)

**Table:**

	*U*^11^	*U*^22^	*U*^33^	*U*^12^	*U*^13^	*U*^23^
N1	0.0416 (16)	0.0425 (14)	0.0471 (15)	−0.0020 (12)	−0.0009 (13)	0.0028 (11)
O1	0.0476 (13)	0.0498 (12)	0.0507 (13)	0.0062 (10)	−0.0107 (10)	0.0102 (10)
O2	0.0382 (12)	0.0539 (12)	0.0468 (12)	0.0001 (9)	−0.0099 (10)	−0.0034 (10)
O3	0.0574 (15)	0.0524 (12)	0.0465 (12)	−0.0163 (11)	−0.0104 (10)	0.0093 (10)
C1	0.0432 (19)	0.0409 (16)	0.0370 (16)	0.0035 (14)	−0.0001 (15)	−0.0002 (12)
C2	0.0364 (17)	0.0391 (15)	0.0367 (16)	0.0038 (13)	−0.0001 (14)	−0.0028 (13)
C3	0.0373 (17)	0.0377 (15)	0.0332 (15)	0.0073 (13)	0.0001 (14)	0.0003 (12)
C4	0.0285 (15)	0.0461 (16)	0.0315 (15)	0.0027 (13)	−0.0040 (13)	−0.0034 (12)
C5	0.0368 (18)	0.0422 (16)	0.0357 (16)	−0.0041 (13)	−0.0011 (14)	0.0002 (13)
C6	0.0488 (19)	0.0471 (17)	0.0328 (16)	−0.0029 (15)	−0.0055 (15)	0.0091 (13)
C7	0.0406 (18)	0.0445 (16)	0.0341 (15)	−0.0010 (14)	−0.0075 (14)	0.0040 (13)
C8	0.198 (5)	0.085 (3)	0.038 (2)	0.004 (3)	0.004 (3)	0.012 (2)
C9	0.062 (2)	0.087 (2)	0.0472 (19)	−0.015 (2)	−0.0120 (18)	−0.0113 (18)
C10	0.102 (3)	0.079 (3)	0.072 (2)	−0.041 (2)	−0.030 (2)	0.036 (2)
C11	0.057 (2)	0.071 (2)	0.057 (2)	−0.0187 (17)	−0.0196 (18)	0.0195 (17)
C12	0.0378 (17)	0.0367 (15)	0.0357 (16)	0.0000 (13)	−0.0035 (14)	−0.0031 (12)
C13	0.0394 (18)	0.0420 (17)	0.0534 (19)	0.0042 (14)	−0.0036 (15)	−0.0067 (13)
C14	0.051 (2)	0.0357 (17)	0.075 (2)	0.0031 (15)	−0.0084 (19)	−0.0022 (15)
C15	0.053 (2)	0.0359 (17)	0.070 (2)	−0.0102 (15)	−0.0104 (18)	0.0042 (15)
C16	0.0410 (18)	0.0396 (16)	0.0462 (17)	−0.0063 (14)	−0.0066 (15)	−0.0045 (13)
C17	0.0373 (17)	0.0382 (16)	0.0369 (16)	−0.0005 (14)	−0.0074 (14)	−0.0061 (12)
C18	0.045 (2)	0.0382 (16)	0.0555 (19)	0.0033 (14)	−0.0038 (16)	−0.0039 (14)
C19	0.044 (2)	0.054 (2)	0.077 (2)	0.0105 (16)	−0.0064 (19)	−0.0060 (17)
C20	0.0356 (19)	0.063 (2)	0.085 (3)	−0.0022 (17)	0.0030 (19)	−0.0095 (19)
C21	0.0411 (19)	0.0558 (19)	0.072 (2)	−0.0134 (16)	−0.0018 (18)	0.0003 (16)

### Geometric parameters (Å, °)

**Table:**

N1—C1	1.279 (4)	C10—H10A	0.96
N1—C12	1.418 (3)	C10—H10B	0.96
O1—C3	1.376 (3)	C10—H10C	0.96
O1—C8	1.395 (4)	C11—H11A	0.96
O2—C4	1.381 (3)	C11—H11B	0.96
O2—C9	1.434 (3)	C11—H11C	0.96
O3—C5	1.371 (3)	C12—C13	1.372 (4)
O3—C10	1.426 (3)	C12—C17	1.434 (4)
C1—C2	1.457 (4)	C13—C14	1.401 (4)
C1—H1	0.93	C13—H13	0.93
C2—C3	1.410 (4)	C14—C15	1.351 (4)
C2—C7	1.416 (4)	C14—H14	0.93
C3—C4	1.393 (4)	C15—C16	1.423 (4)
C4—C5	1.397 (4)	C15—H15	0.93
C5—C6	1.386 (4)	C16—C21	1.417 (4)
C6—C7	1.393 (4)	C16—C17	1.419 (4)
C6—H6	0.93	C17—C18	1.413 (4)
C7—C11	1.505 (4)	C18—C19	1.364 (4)
C8—H8A	0.96	C18—H18	0.93
C8—H8B	0.96	C19—C20	1.390 (4)
C8—H8C	0.96	C19—H19	0.93
C9—H9A	0.96	C20—C21	1.362 (4)
C9—H9B	0.96	C20—H20	0.93
C9—H9C	0.96	C21—H21	0.93
			
C1—N1—C12	117.3 (2)	O3—C10—H10C	109.5
C3—O1—C8	117.1 (2)	H10A—C10—H10C	109.5
C4—O2—C9	114.7 (2)	H10B—C10—H10C	109.5
C5—O3—C10	117.8 (2)	C7—C11—H11A	109.5
N1—C1—C2	126.3 (3)	C7—C11—H11B	109.5
N1—C1—H1	116.9	H11A—C11—H11B	109.5
C2—C1—H1	116.9	C7—C11—H11C	109.5
C3—C2—C7	118.5 (2)	H11A—C11—H11C	109.5
C3—C2—C1	116.6 (2)	H11B—C11—H11C	109.5
C7—C2—C1	124.9 (3)	C13—C12—N1	122.7 (3)
O1—C3—C4	119.1 (2)	C13—C12—C17	119.8 (3)
O1—C3—C2	118.8 (2)	N1—C12—C17	117.4 (2)
C4—C3—C2	122.0 (2)	C12—C13—C14	120.4 (3)
O2—C4—C3	120.2 (2)	C12—C13—H13	119.8
O2—C4—C5	121.0 (2)	C14—C13—H13	119.8
C3—C4—C5	118.6 (2)	C15—C14—C13	121.6 (3)
O3—C5—C6	124.8 (2)	C15—C14—H14	119.2
O3—C5—C4	115.2 (2)	C13—C14—H14	119.2
C6—C5—C4	120.0 (3)	C14—C15—C16	120.0 (3)
C5—C6—C7	122.2 (2)	C14—C15—H15	120.0
C5—C6—H6	118.9	C16—C15—H15	120.0
C7—C6—H6	118.9	C21—C16—C17	118.7 (3)
C6—C7—C2	118.6 (3)	C21—C16—C15	121.9 (3)
C6—C7—C11	118.3 (2)	C17—C16—C15	119.3 (3)
C2—C7—C11	123.0 (3)	C18—C17—C16	118.6 (3)
O1—C8—H8A	109.5	C18—C17—C12	122.8 (3)
O1—C8—H8B	109.5	C16—C17—C12	118.7 (2)
H8A—C8—H8B	109.5	C19—C18—C17	120.8 (3)
O1—C8—H8C	109.5	C19—C18—H18	119.6
H8A—C8—H8C	109.5	C17—C18—H18	119.6
H8B—C8—H8C	109.5	C18—C19—C20	120.6 (3)
O2—C9—H9A	109.5	C18—C19—H19	119.7
O2—C9—H9B	109.5	C20—C19—H19	119.7
H9A—C9—H9B	109.5	C21—C20—C19	120.6 (3)
O2—C9—H9C	109.5	C21—C20—H20	119.7
H9A—C9—H9C	109.5	C19—C20—H20	119.7
H9B—C9—H9C	109.5	C20—C21—C16	120.6 (3)
O3—C10—H10A	109.5	C20—C21—H21	119.7
O3—C10—H10B	109.5	C16—C21—H21	119.7
H10A—C10—H10B	109.5		
			
C12—N1—C1—C2	−179.6 (2)	C1—C2—C7—C6	−177.7 (3)
N1—C1—C2—C3	−171.3 (3)	C3—C2—C7—C11	−178.5 (3)
N1—C1—C2—C7	8.5 (4)	C1—C2—C7—C11	1.7 (4)
C8—O1—C3—C4	−78.3 (4)	C1—N1—C12—C13	47.2 (4)
C8—O1—C3—C2	103.9 (3)	C1—N1—C12—C17	−135.8 (3)
C7—C2—C3—O1	174.0 (2)	N1—C12—C13—C14	179.2 (3)
C1—C2—C3—O1	−6.2 (4)	C17—C12—C13—C14	2.3 (4)
C7—C2—C3—C4	−3.7 (4)	C12—C13—C14—C15	−0.2 (5)
C1—C2—C3—C4	176.1 (2)	C13—C14—C15—C16	−0.2 (5)
C9—O2—C4—C3	109.1 (3)	C14—C15—C16—C21	178.3 (3)
C9—O2—C4—C5	−76.4 (3)	C14—C15—C16—C17	−1.7 (4)
O1—C3—C4—O2	−0.5 (4)	C21—C16—C17—C18	2.8 (4)
C2—C3—C4—O2	177.2 (2)	C15—C16—C17—C18	−177.2 (3)
O1—C3—C4—C5	−175.2 (2)	C21—C16—C17—C12	−176.2 (3)
C2—C3—C4—C5	2.5 (4)	C15—C16—C17—C12	3.8 (4)
C10—O3—C5—C6	2.4 (4)	C13—C12—C17—C18	176.9 (3)
C10—O3—C5—C4	−176.5 (3)	N1—C12—C17—C18	−0.1 (4)
O2—C4—C5—O3	4.6 (4)	C13—C12—C17—C16	−4.1 (4)
C3—C4—C5—O3	179.2 (2)	N1—C12—C17—C16	178.8 (2)
O2—C4—C5—C6	−174.4 (2)	C16—C17—C18—C19	−2.2 (4)
C3—C4—C5—C6	0.2 (4)	C12—C17—C18—C19	176.8 (3)
O3—C5—C6—C7	179.4 (3)	C17—C18—C19—C20	0.1 (5)
C4—C5—C6—C7	−1.7 (4)	C18—C19—C20—C21	1.3 (5)
C5—C6—C7—C2	0.6 (4)	C19—C20—C21—C16	−0.7 (5)
C5—C6—C7—C11	−178.9 (3)	C17—C16—C21—C20	−1.4 (5)
C3—C2—C7—C6	2.1 (4)	C15—C16—C21—C20	178.6 (3)

### Hydrogen-bond geometry (Å, °)

**Table:**

*D*—H···*A*	*D*—H	H···*A*	*D*···*A*	*D*—H···*A*
C13—H13···O3^i^	0.93	2.56	3.489 (4)	178

Symmetry codes: (i) −*x*−1/2, *y*+1/2, *z*.
